# An unusual case of pediatric-type follicular lymphoma: A case report

**DOI:** 10.1097/MD.0000000000017567

**Published:** 2019-10-11

**Authors:** Hanyu Zhang, Shuai Sun, Biru Zhang, Hongyu Yang

**Affiliations:** a Department of Oral and Maxillofacial Surgery, Peking University Shenzhen Hospital, Shenzhen, Guangdong Province; b Graduate Department, Anhui Medical University, Hefei, Anhui Province, P.R. China.

**Keywords:** B-cell lymphoma 2, indolent behavior, pediatric-type follicular lymphoma

## Abstract

**Rationale:**

Pediatric-type follicular lymphoma (PTFL) is a rare neoplasm with features that differ from those of adult-type follicular lymphoma. Compared to patients with adult-type follicular lymphoma, PTFL patients often show an excellent response. Preoperative diagnosis is challenging and, therefore, an accurate diagnosis is based on the findings of postoperative pathological examination and immunohistochemistry.

**Patient concerns:**

A 13-year-old boy presented with a slow-growing mass on the right side of his neck.

**Diagnoses:**

The patient was diagnosed with PTFL based on the findings of histopathological examination and immunohistochemistry.

**Intervention:**

The mass was completely resected.

**Outcomes:**

After 12 months of postoperative follow-up, the patient achieved good recovery without recurrence.

**Lessons:**

The optimal treatment for PTFL has not yet been defined. However, patients with PTFL always show satisfactory prognoses, regardless of treatment strategy (targeted radiotherapy, multiagent chemotherapy, or “watch and wait” strategy). Clinically, pathological and immunohistochemical analyses are necessary in the diagnoses of PTFL cases, especially for distinguishing PTFL from reactive follicular hyperplasia, to avoid unnecessary treatment.

## 1 Introduction

Follicular lymphoma is a type of neoplasm that originates in germinal center (GC)-derived B cells.^[1]^ Follicular lymphoma is commonly observed in adults as a low-grade lymphoma and is always associated with widespread lymphadenopathy. However, albeit rare, when it occurs in children and adolescents, it is often associated with high proliferation rates and is likely to present as localized disease.^[2]^ Pediatric-type follicular lymphoma (PTFL) has distinctive clinical and pathological features. It is a newly recognized variant of follicular lymphoma in the World Health Organization (WHO) Classification.^[3]^ A majority of PTFL patients have been reported to present with localized stage I disease and to demonstrate generally indolent clinical courses, characterized by high-grade cytology, lack of the characteristic t(14;18) (q32;q21) chromosomal translocation, and low B-cell lymphoma 2 (BCL-2) protein expression.^[4]^ PTFL, a distinct variant of adult follicular lymphoma, is typically observed in patients aged between 3 and 18 years, with a male-to-female ratio of approximately 4:1.^[5]^ It occurs most commonly in the cervical lymph nodes and tonsils; however, extranodal occurrence of PTFL has been reported, such as in the testis, gastrointestinal tract, parotid gland, and dura. Unlike follicular lymphoma in adults, PTFL always has an excellent prognosis, with an event-free survival of 90%.^[6]^ Herein, we report the case of a young male patient with PTFL with BCL-2 protein expression.

## 2 Case presentation

**Figure 1:**
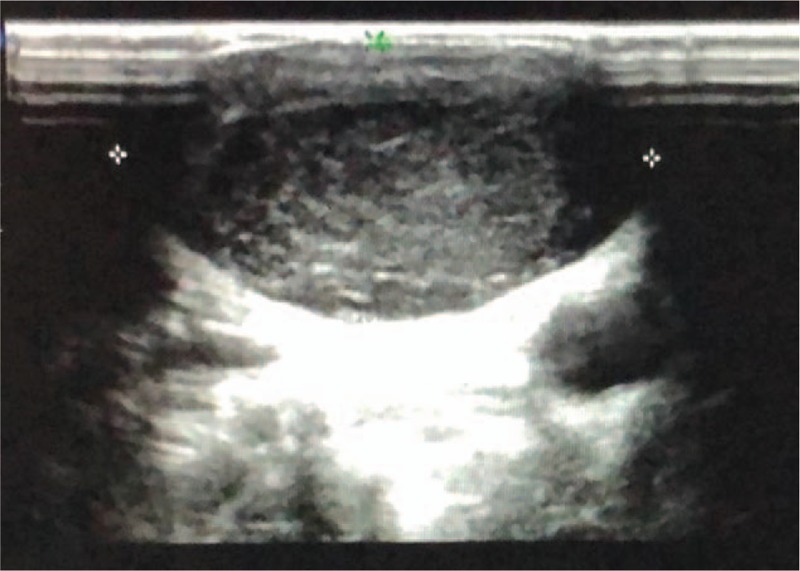
Ultrasound examination revealed a well-circumscribed hypoechogenic image of the right neck.

A 13-year-old boy presented to our hospital with a 3 cm-sized mass in the right cervical region. The mass was painless without any inflammatory features. He had no recent history of illness or fever. He had undergone appendectomy at the age of 8 years. Clinical examination revealed a 3.5 × 2 cm-sized mass at the superior border of the right sternocleidomastoid muscle; the mass had medium texture, a clear movable boundary, was nontender, and did not adhere to the surrounding tissues. No other cervical masses were palpated. Ultrasound examination performed at another hospital had shown an abnormal acoustic image of approximately 3 cm in the right cervical region at level II (Fig. 1). No enlarged lymph nodes were observed near the large vessels in the lateral aspect of the neck. He did not undergo computed tomography or magnetic resonance imaging at our hospital. Based on his clinical symptoms and specialist examination, he was initially diagnosed with a mass in the right neck. Chest radiography and laboratory tests, such as complete blood cell count and coagulation studies, showed unremarkable results.

A 5 cm incision was made during surgery, allowing complete exposure of the mass; the mass showed no adhesion to the surrounding tissue and a clear boundary. The tumor was then completely excised. Examination of an intraoperative frozen section revealed lymphoid tissue hyperplasia.

**Figure 2:**
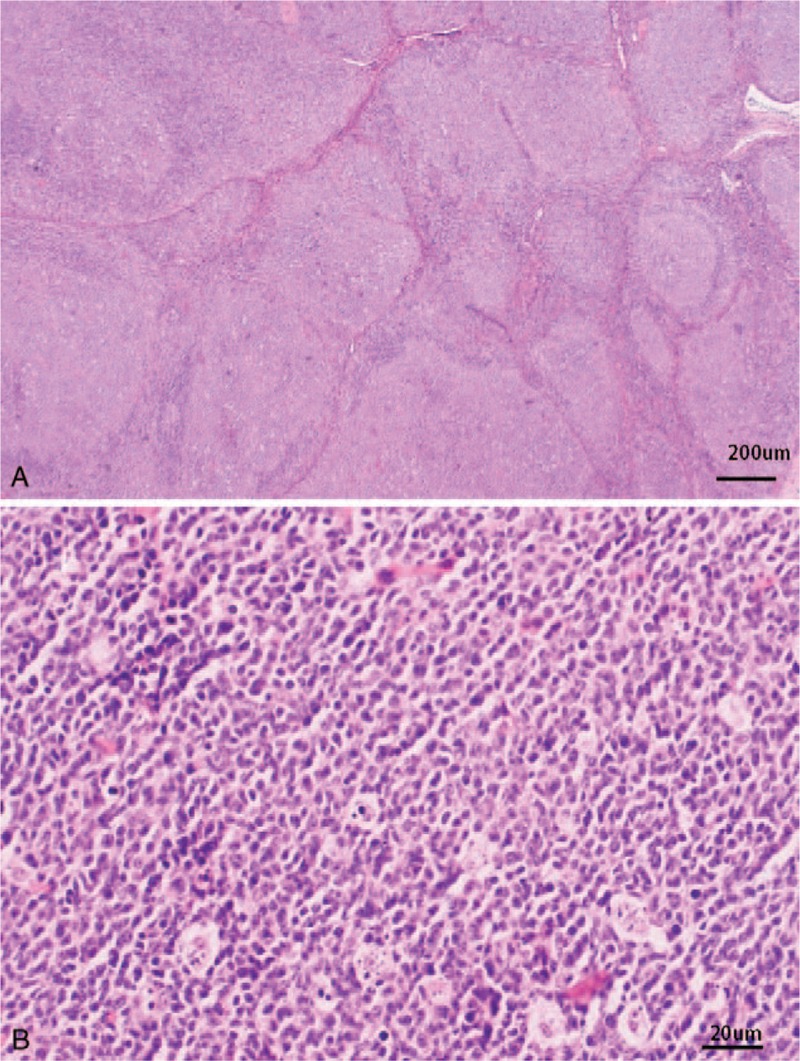
Histopathological features of pediatric-type follicular lymphoma. A, Large and irregular lymphoid follicles were densely distributed, a starry-sky pattern is evident (H&E, ×40). B, Composed of medium-sized centrocytes and numerous medium-sized centroblasts (H&E, ×400).

**Figure 3:**
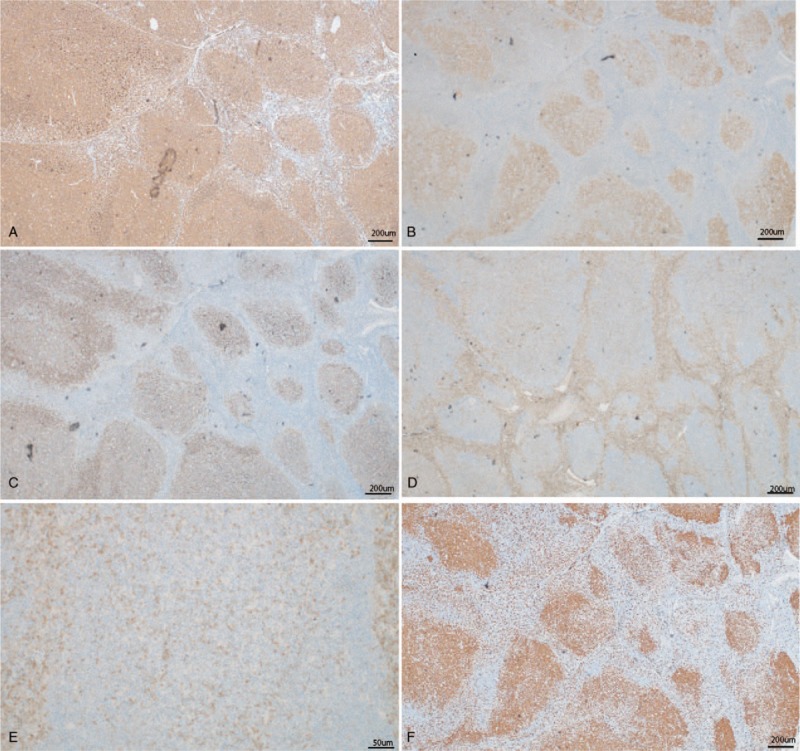
Immunophenotypic features of pediatric follicular lymphoma. A, Immunohistochemistry for CD20 highlights the follicular architecture of this tumor (×40). B, The neoplastic cells is positive for BCL-6 (×40). C, Irregular expanded networks of follicular dendritic cells as highlighted in this immunostain for CD21 (×40). D, Immunostaining reveals partial expression of bcl-2 by neoplastic lymphoma cells within the nodules (×40). E, Higher-power image of BCL2 (×200). F, The Ki-67 proliferative index in the follicular area, at approximately 90% (×40).

Immunohistochemistry and molecular pathology were performed to further differentiate the diagnosis from that of various lymphoproliferative or neoplastic lesions. Microscopically, the specimen showed hyperplasia of lymphoid tissue with destruction of nodal architecture and a densely arranged follicular structure, with partial follicular expansion or structural destruction and typically thin or disappearing mantle zones (Fig. 2). Immunohistochemical studies revealed that CD20 expression (B lymphocytes) was strongly positive in lymphoid follicles (Fig. 3A); staining was positive for CD10, CD3+ (T lymphocytes), and BCL-6 (Fig. 3B); CD21 staining showed irregular expanded networks of follicular dendritic cells (Fig. 3C); some of the germinal-center B cells expressed BCL-2 protein (Fig. 3D, E). The Ki-67 labeling index in GCs exceeded 90% (Fig. 3F). The specimen tested negative for CD30, CD38, MUM-1, cyclin D1, TdT, kappa, and lambda. On fluorescence in situ hybridization, no rearrangement was detected in the *BCL2/BCL6* genes; however, a monoclonal rearrangement was detected in the immunoglobulin heavy chain gene. The patient was eventually diagnosed with PTFL without margin involvement. Postoperatively, the patient refused any further local or systemic treatment. No evidence of recurrence has been observed after 12 months of follow-up.

## 3 Discussion

Here, we described a case of follicular lymphoma in a 13-year-old male patient. PTFL is designated as a separate entity from follicular lymphoma according to the WHO classification.^[3]^ It is an extremely rare disease and predominantly occurs in males as localized stage I disease and follows an indolent clinical course.^[7]^ PTFL is not restricted to patients in the age group of children. PTFL has been reported to occur frequently in young adults between the ages of 18 and 30 years; it occasionally occurs in older adults with the same indolent behavior.^[4,8–10]^

The morphological features of PTFL are similar to those of adult-PTFL. PTFL is always characterized by high-grade histology, lack of the t(14;18) (q32;q21) chromosomal translocation, and weak or negative BCL-2 protein expression.^[8]^ In this case, immunohistochemistry showed partial BCL-2 expression in GC-derived B-cells. Lorsbach et al^[7]^ reported that approximately 30% of PTFL cases were positive for BCL-2, suggesting that BCL-2 expression in PTFL may have prognostic significance. Oschlies et al^[11]^ suggested that BCL-2-positive PTFL showed a trend toward high disease stage, indicating aggressive tumor growth. Louissaint et al^[12]^ found that BCL-2 protein expression in PTFL patients may indicate aggressive disease or indicate that the disease is highly refractory to chemotherapy.

Despite the high frequency of aggressive cytological features, PTFL patients show excellent prognoses to surgical resection or multiagent chemotherapy, and the recurrence rate is extremely low. It has been reported that the probability of 5-year event-free survival was >90%.^[11]^ This characteristic was well reflected in our patient, who showed good recovery with no recurrence without any chemotherapy after the 12-month follow-up. Originally, PTFL patients were often treated with chemotherapy.^[13]^ In recent years, a variety of treatments have been used, such as “watch and wait” strategy, targeted radiotherapy, and multiagent chemotherapy.^[6,14,15]^

The diagnosis of PTFL can be complicated by the presence of clinical and morphological features that overlap with those of reactive follicular hyperplasia or pediatric nodal marginal zone lymphoma (PNMZL). Preoperative examination, such as B-mode ultrasound or magnetic resonance imaging is helpful; however, the final diagnosis depends on the histological and molecular characteristics. The identification of these diseases is crucial to clinical treatment and prognosis. In contrast to reactive follicular hyperplasia cases, most cases of PTFL show irregularly shaped follicles, attenuated mantle zones, and high levels of proliferation-based markers, like the Ki-67 index.^[14]^ An immunophenotypic study suggested that GC-associated markers, like BCL-6 and CD10, seen in PTFL are absent in PNMZL.^[16]^ Furthermore, in PNMZL, the interfollicular areas typically show low Ki-67 proliferation rates.^[8]^

The genetic alterations involved in the pathogenesis of PTFL are yet to be elucidated. Recently, studies have shown that the *TNFRSF14* and *MAP2K1* genes are frequently mutated in PTFL, suggesting that both mutations might play an important role in PTFL lymphomagenesis.^[9,10,17]^

Since the optimal clinical management is yet to be defined, our report adds value to the literature as it suggests that a “watch and wait” strategy can be a good option. Compared with other malignant tumors, PTFL patients always have excellent prognoses. However, many factors related to PTFL remain to be elucidated, especially regarding its molecular pathology and etiopathogenesis; with this knowledge, we can accurately diagnose and effectively treat this disease.

## Author contributions

**Conceptualization:** Hongyu Yang.

**Funding acquisition:** Hongyu Yang.

**Investigation:** Biru Zhang.

**Software:** Biru Zhang.

**Supervision:** Shuai Sun.

**Writing – original draft:** Hanyu Zhang.

**Writing – review and editing:** Hanyu Zhang.
